# Neonatal Screening for Congenital Adrenal Hyperplasia in Guangzhou: 7 Years of Experience

**DOI:** 10.3390/ijns11040116

**Published:** 2025-12-17

**Authors:** Xuefang Jia, Ting Xie, Xiang Jiang, Fang Tang, Minyi Tan, Qianyu Chen, Sichi Liu, Yonglan Huang, Li Tao

**Affiliations:** Department of Guangzhou Newborn Screening Center, Guangzhou Women and Children’s Medical Center, Guangzhou Medical University, Guangzhou 510180, China; jiaxuefang8888@163.com (X.J.);

**Keywords:** 21-hydroxylase deficiency, congenital adrenal hyperplasia, 17-hydroxyprogesterone, gestational age, collection time, newborn screening

## Abstract

This study was designed to assess the effectiveness of neonatal congenital adrenal hyperplasia (CAH) screening in Guangzhou, China. A total of 818,417 newborns were screened for CAH by measuring 17-hydroxyprogesterone (17-OHP) concentrations. Cut-off values were stratified based on gestational age (GA) and the timing of sample collection. Neonates with initial positive results (17-OHP ≥ cut-off value) were recalled for a second dried blood spot sample to reassess 17-OHP levels. Confirmatory testing involved biochemical analyses, Sanger sequencing, and multiplex ligation-dependent probe amplification of the *CYP21A2* gene. From 2018 to 2024, a total of 40 patients with classical 21-hydroxylase deficiency were identified, including 28 cases (70%) of the salt-wasting form and 12 cases (30%) of the simple virilizing form. The overall incidence of CAH was 1 in 20,653 (95% confidence interval: 1:34,928, 1:14,661). No statistically significant differences in prevalence were observed between sexes or between preterm and full-term infants (*p* > 0.05). 17-OHP concentrations are influenced by GA and the timing of sample collection. The screening efficiency for CAH could be improved by adopting a multitiered cut-off value system adjusted for GA and collection time.

## 1. Introduction

Congenital adrenal hyperplasia (CAH) constitutes a common autosomal recessive disorder resulting from genetic mutations in enzymes essential for cortisol biosynthesis. This adrenal dysfunction presents with diverse clinical manifestations, ranging from life-threatening salt-wasting crises to virilization. CAH is classified into classical form and non-classical (NC) form. Classical CAH is further categorized into salt-wasting (SW) form and simple virilizing (SV) form. Although pathogenic variants have been identified in several genes, including *CYP11B1*, *CYP17A1*, *HSD3B2*, *POR*, *STAR*, and *CYP11A1*, approximately 95% of CAH cases are caused by 21-hydroxylase deficiency (21-OHD), primarily due to mutations in the *CYP21A2* gene [1,2]. Epidemiological data indicate that the global prevalence of 21-OHD ranges from 1 in 10,000 to 1 in 20,000 live births [3,4,5].

Early diagnosis and timely therapeutic intervention are crucial for reducing the morbidity and mortality associated with CAH. Newborn screening (NBS) programs have become a cornerstone for the early detection of CAH, primarily through the measurement of 17-hydroxyprogesterone (17-OHP), a precursor metabolite that accumulates due to CYP21A2 enzyme deficiency. The automated dissociation-enhanced lanthanide fluorescence immunoassay (DELFIA) system is widely utilized for 17-OHP quantification. However, elevated 17-OHP levels in preterm neonates often lead to false-positive results, thereby compromising the specificity of the screening process [3,6].

The primary objective of neonatal screening for CAH in Guangzhou is the early detection of classical 21-OHD. This study presents a comprehensive analysis of seven years of longitudinal data from the 21-OHD newborn screening program in Guangzhou, China, with two principal aims: (1) to evaluate the effectiveness of the current screening protocol and (2) to establish optimal 17-OHP cutoff thresholds to minimize false-positive results while improving the positive predictive value (PPV). Meanwhile, our study incorporated clinical data and genetic testing results from a subset of patients with confirmed CAH, with the aim of enabling a more comprehensive evaluation of neonatal screening effectiveness.

## 2. Subjects and Methods

### 2.1. Subjects

Dried blood spot (DBS) samples were systematically collected from over 100 delivery hospitals in Guangzhou and subsequently transported to the laboratory via standard postal services. Data extracted from the screening program database included sex, GA, birth weight (BW), and collection time. Prior to blood sample collection, informed consent was obtained from the parents or legal guardians of the neonates. The study protocol received ethical approval from the Ethics Committee of Guangzhou Women and Children’s Medical Center.

### 2.2. CAH Newborn Screening Program

**Figure 1:**
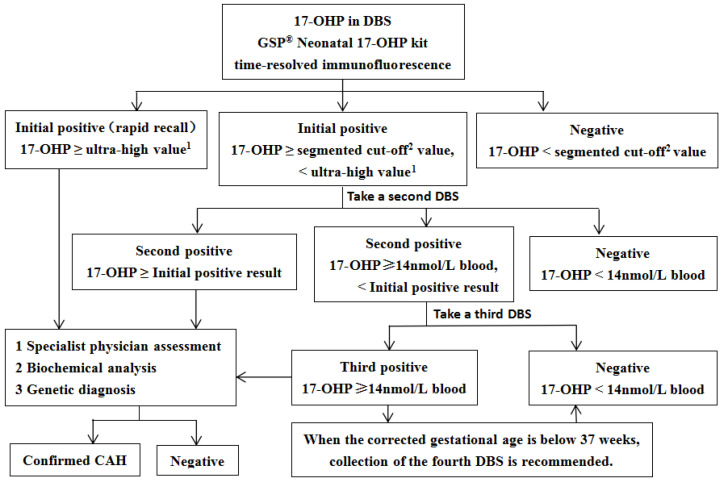
**The flowchart of CAH screening in Guangzhou.** ^1^ preterm birth: 150 nmol/L blood; full-term birth: 100 nmol/L blood; ^2^ Preterm birth: 60 nmol/L blood (<32 weeks), 35 nmol/L blood (32–36 weeks), 19 nmol/L blood (36–37 weeks); full-term birth (≥37 weeks): 19 nmol/L blood (24–48 h), 14 nmol/L blood (≥48 h); For preterm infants with positive initial screening results, the following re-examination protocol is recommended: perform DBS 17-OHP testing every 2 to 3 weeks, or complete re-examination at 37 weeks of corrected gestational age or prior to discharge, following specialist evaluation.17-OHP, 17-hydroxyprogesterone; DBS, dried blood spot; GSP, genetic screening processor; CAH, congenital adrenal hyperplasia.

The concentration of 17-OHP in 3.2 mm DBS was measured using the Genetic Screening Processor (GSP^®^ Neonatal 17-OHP kit, Wallac Oy, Turku, Finland) based on a time-resolved immunofluorescence assay. As illustrated in Figure 1, the initial 17-OHP screening employs a segmented cutoff approach. Newborns with significantly elevated initial screening results require immediate recall for comprehensive specialist evaluation. Those with positive results below the extreme threshold undergo second or third DBS collection for retesting. Persistently positive retest results necessitate further biochemical and genetic diagnostic investigations. Biochemical analysis includes serum sodium, serum potassium, 17-hydroxyprogesterone, progesterone, adrenocorticotropic hormone, cortisol, androstenedione, testosterone, and dehydroepiandrosterone sulfate. Genetic diagnosis of the *CYP21A2* gene was conducted using targeted amplification, followed by Sanger sequencing and multiplex ligation-dependent probe amplification (MLPA). The SV form in females is characterized by virilization of the external genitalia. When accompanied by electrolyte disturbances, specifically hyponatremia and hyperkalemia, it is classified as SW form. Some of the initially positive screening specimens were included in a pilot study of liquid chromatography-tandem mass spectrometry (LC-MS/MS) second-tier screening and were therefore not included in the flowchart (Figure 1).

In 2018, we measured 17-OHP levels in approximately 50,000 samples and established segmented cut-off values based on the 99.5th percentile, stratified by GA and collection time, as detailed in Figure 1.

In this study, a retrospective analysis was conducted using historical data from 2018 to 2024, leading to the establishment of a new cut-off value based on the 99.5th percentile. This newly defined cut-off was subsequently applied to the corresponding dataset, and a systematic comparison was carried out between the performance and impact of the new and existing cut-off values in practical application, with the aim of evaluating their respective applicability and effectiveness.

### 2.3. Statistical Analysis

Statistical analyses were conducted using SPSS version 26.0. The normality of data distribution was assessed using the Kolmogorov-Smirnov one-sample test. The 99.5th percentile was determined to establish the threshold value. Intergroup comparisons in rates were performed using the chi-square test, with statistical significance defined as *p* < 0.05. The PPV was calculated using the following formula: 100% × (number of confirmed cases/number of initially screen-positive cases). In this study, individuals with a positive result on the initial DBS test were classified as screen-positive.

## 3. Results

### 3.1. The Incidence and Clinical Manifestations of CAH

This program included all newborns born in Guangzhou between February 2018 and December 2024 who underwent screening for CAH. A government-funded free CAH neonatal screening program was implemented in Guangzhou on 1 June 2021, thereby ensuring universal coverage for all newborns born in the city thereafter. The study encompassed a total of 818,417 newborns, with 438,819 (53.6%) identified as male and 379,598 (46.4%) identified as female. Of these, 54,848 (6.7%) were classified as preterm births.

Over a seven-year period, this study identified a total of 53 cases of CAH. As detailed in Table S2, ten cases were not subjected to neonatal CAH screening, and three cases were outside the target scope of the screening program—specifically, two cases of NC form and one case of 3β-hydroxysteroid dehydrogenase deficiency. These 13 cases were excluded from subsequent data analysis.

Among the 40 infants diagnosed with 21-OHD via newborn screening and classified as classical forms (excluding non-classical cases), 20 were male and 20 were female, representing an equal distribution of 50% each. The sex-specific incidence rates were determined to be 1 in 18,980 (95% confidence interval [CI]: 1:33,784, 1:13,196) for females (20/379,598) and 1 in 21,941 (95% CI: 1:39,063, 1:15,256) for males (20/438,819), with no statistically significant difference in prevalence observed between sexes (*p* > 0.05). The overall incidence of CAH in neonates between 2022 and 2024 was 1 in 20,653 (95% CI: 1:34,928, 1:14,661), with annual rates ranging from 1 in 15,431 to 1 in 52,369 (Table 1), demonstrating no significant interannual variability (*p* > 0.05). Among the CAH cases, preterm births constituted 7.5% (3/40), corresponding to a prematurity-specific incidence of 1 in 18,283 (3/54,848; 95% CI: 0, 1:8577), which did not significantly differ from the incidence in term infants (*p* > 0.05).

Among the 40 patients, 26 cases (65%) had already exhibited varying degrees of clinical manifestations by the time the screening results were available, including skin and genital pigmentation, clitoral hypertrophy, and external genital malformations. As detailed in Table S1, in this clinical cohort of 40 patients diagnosed with CAH, phenotypic analysis revealed a distribution of 28 cases (70%) exhibiting the SW form, 12 cases (30%) presenting the SV form. A familial predisposition to CAH was observed in four patients. Among the 20 female patients, 10 (50%) were diagnosed neonatally, primarily due to the presence of ambiguous genitalia.

As detailed in Table S2, among the ten patients who did not undergo neonatal CAH screening, nine presented with the SW form and one with the SV form, the latter having a family history of the condition; all seven female patients exhibited clinical manifestations of ambiguous genitalia.

### 3.2. Analysis of CYP21A2 Gene Mutations

As presented in Table 2, comprehensive genetic analysis of 36 patients revealed 14 distinct variants in the *CYP21A2* gene, with p.Ser97fs*12 emerging as the most prevalent mutation. As presented in Table S1, Homozygosity for p.Ser97fs*12 was identified in seven patients, six of whom presented with the SW phenotype, while one exhibited the SV phenotype. Among the 11 individuals heterozygous for the p.Ser97fs*12 variant, eight manifested the SW phenotype. Large deletions, defined as the deletion of more than one exon, were detected in nine cases, six of which exhibited the SW phenotype. The p.Ile173Asn mutation was associated with the SW phenotype in 7 out of 15 cases, whereas the p.Arg357Trp mutation resulted in SW in 4 out of 5 cases.

As presented in Table S2, the two patients (Cases 11 and 12) with the NC form were genotyped as p.Ser97fs*12/c.-113G>A and p.Ser97fs*12/p.Val282Leu, respectively. Additionally, one male patient (Cases 13) with hypospadias was diagnosed with 3β-hydroxysteroid dehydrogenase deficiency (p.Thr259Met/p.Val225Asp) and classified as SW.

### 3.3. False-Positive Cases

As presented in Table 3, the implementation of existing diagnostic threshold criteria during the study period yielded 3064 false-positive cases, corresponding to a false-positive rate of 0.37%, with an overall PPV of 1.3%. Notably, preterm infants exhibited a significantly elevated false-positive rate (1.76–3.40%) compared to full-term infants (0.23–0.25%; *p* < 0.05). Furthermore, the PPV in preterm infants ranged from 0% to 0.4%, which was markedly lower than the 1.8–2.1% observed in full-term infants (*p* < 0.05).

To enhance the screening efficacy for preterm neonates, we conducted a comprehensive reanalysis of the percentile distribution of 17-OHP concentrations across multiple tiers stratified by GA and collection time. Revised cut-off values were determined based on the 99.5th percentile of 17-OHP concentrations, as detailed in Table 4. The updated cutoff values were applied to the same dataset for retrospective analysis. The results demonstrated that the false positive rate among preterm infants decreased to 0.51–0.68%, while the PPV increased to 0–1.2%. These findings are summarized in Table 3. In addition, between 2018 and 2021, the annual false positive rate for neonatal screening of CAH ranged from 0.24% to 0.37%. Following the full implementation of neonatal CAH screening across Guangzhou starting in 2022, the false positive rate has stabilized at approximately 0.4%, as shown in Table 1.

### 3.4. False-Negative Cases

Among the neonates ultimately diagnosed with CAH, one female infant (Case No. 30, Table S1) was nearly misdiagnosed. Initial newborn screening revealed a 17-OHP level of 17.2 nmol/L blood, measured from a DBS sample collected between 24–48 h postnatally. Application of the standard 19 nmol/L blood cut-off threshold would have resulted in a missed diagnosis; however, the identification of a positive family history for CAH led to the infant’s recall and subsequent confirmatory testing.

### 3.5. Establishment and Application of New 17OHP Concentration Cutoffs

As shown in Table 4, the 99.5th percentile values of 17-OHP concentrations in full-term infants vary across the specified sampling intervals. In contrast, these values exhibit relative stability within preterm subgroups.

A total of 53 patients with CAH were diagnosed in this study. These included 40 cases of classic 21-OHD identified through neonatal CAH screening, as detailed in Table S1; ten cases diagnosed without undergoing neonatal screening, as documented in Table S2; and an additional two cases of NC 21-OHD and one case of 3β-hydroxysteroid dehydrogenase deficiency. Among these 53 patients, 36 were full-term infants with blood samples collected at or beyond 48 h after birth, exhibiting 17-OHP concentrations ranging from 15.2 nmol/L to over 600 nmol/L. Additionally, 13 full-term infants sampled between 24–48 h post-birth were confirmed with CAH, demonstrating 17-OHP levels spanning 17.2 nmol/L blood to 564.7 nmol/L blood. No CAH cases were identified among early preterm infants. Among middle preterm and late preterm infants, three and one case(s) were confirmed, respectively, with the lowest 17-OHP concentrations recorded at 192.5 nmol/L blood and 84.2 nmol/L blood.

Multivariable analysis of 17-OHP distributions at the 99.5th percentile, in conjunction with the minimum 17-OHP concentrations identified in confirmed cases of CAH, enabled the establishment of refined diagnostic thresholds stratified by GA and postnatal sampling time. To enhance practicality and ease of recall in clinical settings, the new cutoff values have been rounded to whole numbers and refined based on the data presented in Table 4. The revised thresholds are as follows: for preterm neonates, <32 weeks: 110 nmol/L blood; 32–36 weeks: 50 nmol/L blood; and 36–37 weeks: 25 nmol/L blood. For full-term neonates (≥37 weeks), the thresholds are 17 nmol/L blood for samples collected 24–48 h postnatally and 14 nmol/L blood for samples collected ≥48 h postnatally.

## 4. Discussion

### 4.1. Regional Variations in CAH Incidence Rates and Methodological Considerations in Screening

The incidence of CAH identified through neonatal screening may exhibit minor regional variations, influenced by demographic and methodological factors. In this study, the overall CAH incidence from 2022 to 2024 was 1:20,653, which is comparable to the national average in China (1:23,024) [7] and similar to reported rates in the United States (1:16,825) [8], Brazil (1:15,887) [9], Denmark (1:14,129) [10], and Sweden (1:11,200) [11]. It should be noted, however, that differences in incidence across regions have not been subjected to formal statistical analysis. Methodological variations in screening protocols, particularly in cut-off values and sampling timing across different regions, significantly impact detection outcomes [8]. Research by Nazaneen Eshragh et al. [12] highlights that initial blood sampling within 24–48 h post-birth may result in a 25% false-negative rate for mild CAH cases, underscoring the importance of implementing secondary blood sampling between 10–14 days after birth. Furthermore, specialized screening protocols involving multiple blood samplings have been adopted in numerous countries for specific populations, including preterm infants, low birth weight neonates, critically ill infants, and those exposed to prenatal glucocorticoid therapy [13]. To mitigate incidence rate discrepancies attributable to methodological variations, the standardization of regional screening protocols represents a critical future direction.

### 4.2. Strategies and Constraints in Threshold Optimization

The optimization of 17OHP cut-off values represents a critical strategy for enhancing CAH newborn screening, particularly in preterm, low birth weight, and critically ill neonates. Tailoring these cut-off values to local population characteristics not only improves screening efficacy but also mitigates false-positive rates and reduces unnecessary follow-up interventions. In this study, cut-off values were established based on GA and precise timing of blood collection, resulting in a 68.8% to 84.9% reduction in false-positive rates among preterm infants, consistent with findings from diverse regions, including Brazil [9,14]. Due to the limited sample size in the group with GA ≥42 weeks in Table 4 (*n* = 319), the results may be subject to statistical bias. Therefore, during the subsequent determination of the cutoff value, this group was not analyzed separately but was instead combined with the full-term infant group (37–42 weeks) for analysis. Segmented cut-off values based on blood collection time were not established for preterm infants, as the data demonstrated that variations in 17-OHP levels across different blood collection time points among preterm infants were relatively minor (see Table 4). This approach effectively addresses physiological 17OHP fluctuations attributable to immature adrenal function in preterm populations. However, limitations such as insufficient sample sizes in preterm cohorts may introduce cut-off value deviations, underscoring the need for multicenter collaborations or longitudinal data supplementation to refine these thresholds. Additionally, imprecise documentation of blood collection timing (e.g., “24–48 h” without hourly specificity) may compromise cut-off value efficacy, necessitating stringent temporal management protocols. While various methodologies have been employed globally to optimize 17OHP cut-off values, their impact on improving positive predictive value remains constrained. The integration of LC-MS/MS as a second-tier assay has been widely demonstrated to significantly reduce false-positive rates, enhance screening accuracy and efficiency, and facilitate the identification of rare CAH variants [15,16,17].

### 4.3. Analysis of False Negative Cases

This study identified a false-negative case (Case No. 30, Table S1) with an initial 17OHP level of 17.2 nmol/L blood, which was below the original cut-off value of 19 nmol/L blood for full-term infants within the 24–48-h postnatal window, resulting in a missed diagnosis. To prevent such missed diagnoses, we propose setting the cut-off value at 17 nmol/L of blood, based on a re-evaluation of the 99.5th percentile (see Table 4) and in alignment with the optimized thresholds presented in Table 3. This adjustment improves the clinical utility of the screening program by balancing diagnostic sensitivity with specificity, thereby enhancing both reliability and operational feasibility.

### 4.4. Types and Frequencies of Gene Mutations in CAH

Global investigations into classic 21-OHD have demonstrated that while the distribution between SW and SV forms remains consistent across populations, significant interethnic variations exist in the prevalence of specific mutations, particularly the p.Ser97fs*12 point mutation and large deletion events [18]. Epidemiological data reveal that the p.Ser97fs*12 mutation exhibits higher frequency in populations from central and southern China and Southeast Asia [19,20], whereas in northern China, the p.Ile173Asn mutation predominates, likely due to the increased representation of SV cases in study cohorts [21]. Our findings corroborate previous reports [22] from southern China, with p.Ser97fs*12 emerging as the most prevalent mutation (33.3%). Among the 36 patients with classical 21-OHD listed in Table 2, 70% exhibited the SW phenotype, characterized by a mutational spectrum predominantly composed of the p.Ser97fs*12 variant (20.0%), p.Ile173Asn (8.0%), and large deletions (7.0%). The remaining 30% presented with the SV phenotype, which was primarily associated with the p.Ile173Asn variant (34.8%). The V282L point mutation, primarily linked to non-classic 21-OHD [18,23], demonstrated consistent patterns in our cohort (see Case No. 12, Table S2).

Newborn screening programs are inherently limited and can typically detect only those conditions or variants for which they are specifically designed. Furthermore, the identification of 3β-HSD deficiency cases (p.T259M/p.V225D) highlights the necessity of expanding genetic diagnostic protocols in patients presenting with typical SW phenotypes to detect rare CAH subtypes. Our analysis confirms the conservation of genotype-phenotype correlations across diverse ethnic populations (e.g., severe mutations predominantly resulting in SW phenotypes), while emphasizing the substantial influence of population-specific genetic backgrounds on variant frequencies. These observations underscore the critical need for establishing population-specific genetic databases to enhance the efficacy of newborn screening programs.

## 5. Conclusions

In neonatal screening programs, optimizing the CAH neonatal screening protocol to minimize both false positive and false negative outcomes has long been acknowledged as a central objective within the field. Adjusting cut-off values for preterm infants is regarded as a potentially valuable strategy for enhancing screening specificity. This study established new cut-off criteria specifically designed to mitigate the risks of both false positive and false negative results. However, the PPV observed in this study remains relatively low compared to other regions utilizing the same assay kit (typically <10%) [8,24]. Given current technical limitations, further efforts are warranted to develop more effective screening strategies to enhance overall screening performance.

## Figures and Tables

**Table 1:** Annual incidence rate of CAH.

		Positive	Confirmed		FP	Unscreened	CAH	Low	High
Year	*n*	Cases (*n*)	Cases (*n*)	PPV (%)	Rate (%)	CAH (*n*)	Incidence	95% CI	95% CI
2018	58,076	216	3	1.4%	0.37%	2	/	/	/
2019	78,665	189	3	1.6%	0.24%	5	/	/	/
2020	72,617	189	6	3.2%	0.25%	3	/	/	/
2021	134,051	507	5	1.0%	0.37%	0	/	/	/
2022	157,108	683	3	0.4%	0.43%	1 ^NC^	1/52,369	0	1/24,570
2023	148,162	592	9	1.5%	0.39%	0	1/16,462	1/47,619	1/9958
2024	169,738	731	11	1.6%	0.42%	1 ^NC^; 1 ^3β^	1/15,431	1/37,722	1/9699
Total *	475,008	2006	23	1.2%	0.42%	3	1/20,653	1/34,928	1/14,661

PPV, positive predictive value; FP, false positive; CI, confidence interval; /, From 2018 to 2021, universal screening for CAH had not yet been implemented for newborns in Guangzhou, and therefore, no incidence data were collected during this period; ^NC^, non-classical; The two NC cases presented in the table were confirmed via a positive outcome from LC-MS/MS-based secondary screening and are not included in the incidence rate calculations; ^3β^, 3β-hydroxysteroid dehydrogenase deficiency; *, The data range represented in the “Total” row spans from 2022 to 2024; There was no statistically significant difference in the annual incidence rates of CAH across different years (*p* > 0.05).

**Table 2:** Frequency of Major CYP21A2 Variants in 36 ** Genotyped Patients with Classical 21-OHD.

Variant	Alleles (*n*)	Allele Frequency	Alleles (*n*, SW-CAH)	Allele Frequency (SW-CAH)	Alleles (*n*, SV-CAH)	Allele Frequency (SV-CAH)
p.Ser97fs*12	25	33.3%	20	38.5%	5	21.7%
p.Ile173Asn	16	21.3%	8	15.4%	8	34.8%
Large deletions ^#^	10	13.3%	7	13.5%	3	13.0%
p.Arg357Trp	6	8.0%	5	9.6%	1	4.3%
p.Gln319*	3	4.0%	3	5.8%	0	0.0%
p.Gly111Valfs*21	3	4.0%	2	3.8%	1	4.3%
p.Arg484Profs*58	3	4.0%	3	5.8%	0	0.0%
p.G425S	2	2.7%	0	0.0%	2	8.7%
p.Leu308Phefs*6	2	2.7%	2	3.8%	0	0.0%
Other variants ^##^	5	6.7%	2	3.8%	3	13.0%
Total	75	100.00%	52	100.00%	23	100.00%

21-OHD, 21-hydroxylase deficiency; SW, salt-wasting; SV, simple virilizing; ^#^, deletion of more than one exon. ^##^, 5 alleles comprised 5 other variants (1.3% each). **, A total of 53 patients with CAH were diagnosed in this study. Ten patients who did not undergo neonatal CAH screening, two patients with nonclassical CAH, and one patient with 3β-hydroxysteroid dehydrogenase deficiency were excluded from the analysis. Additionally, genetic testing results were unavailable for four patients. Therefore, the analysis presented in this table includes only the 36 patients with classical 21-OHD.

**Table 3:** Current and proposed reference 17-OHP values (nmol/L blood) in the study population.

	Age at		Current Reference Values					Proposed Reference Values				
	Collection		17-OHP	Positive	Confirmed		FP	17-OHP	Positive	Confirmed		FP
GA (Weeks)	Time (h)	*n*	(nmol/L)	Cases (*n*)	Cases * (*n*)	PPV (%)	Rate (%)	(nmol/L)	Cases (*n*)	Cases * (*n*)	PPV (%)	Rate (%)
<32		5845	60	199	0	0.0%	3.40%	110	30	0	0.0%	0.51%
32–36		24,594	35	541	2	0.4%	2.19%	50	169	2	1.2%	0.68%
36–37		24,409	19	430	1	0.2%	1.76%	25	134	1	0.7%	0.54%
≥37	24–48	201,659	19	481	10	2.1%	0.23%	17	901	11	1.2%	0.44%
	≥48	561,910	14	1452	26	1.8%	0.25%	14	1452	26	1.8%	0.25%
Total		818,417	-	3103	39	1.3%	0.37%	-	2686	40	1.5%	0.32%

GA, gestational age; -, not calculated. *, Ten patients who did not undergo neonatal CAH screening, two patients with nonclassical CAH, and one patient with 3β-hydroxysteroid dehydrogenase deficiency were excluded from the analysis.

**Table 4:** Percentile values (nmol/L blood) for 17-OHP in relation to gestational age (GA) and collection time.

GA, Weeks	Age at Collection Time, h									
	Total		24–48		48–72		72–168		≥168	
	*n*	99.5th	*n_*1*_*	99.5th	*n_*2*_*	99.5th	*n_*3*_*	99.5th	*n_*4*_*	99.5th
<32	5845	112.5	192	134.1	932	100.1	2399	120.5	2322	111.1
32–36	24,594	53.9	2897	53.4	8461	55.5	10,789	53.4	2447	44.7
36–37	24,409	25.2	5009	28.8	9732	24.3	8994	23.5	674	27.9
37–42	763,250	14	201,600	16.5	326,832	12.8	227,755	12	7063	14.7
≥42	319	12.7	59	/	127	11	128	10.9	5	/

/, Sample size too small for statistical analysis.

## Data Availability

Data will be made available to qualified researchers on request.

## Supplementary Materials

The following supporting information can be downloaded at: https://www.mdpi.com/article/10.3390/ijns11040116/s1, Table S1: Clinical characteristics, laboratory findings, and genotype of CAH confirmed cases (forty neonates diagnosed with classic 21-hydroxylase deficiency through newborn screening for CAH). Table S2: Clinical characteristics, laboratory findings, and genotype of CAH confirmed cases (ten cases of children who did not undergo neonatal CAH screening and three cases of children who were not within the target population for screening).

## Abbreviations

CAH, congenital adrenal hyperplasia; NC, non-classical; SW, salt-wasting; SV, simple virilizing; 21-OHD, 21-hydroxylase deficiency; NBS, newborn screening; 17-OHP, 17-hydroxyprogesterone; DELFIA, dissociation-enhanced lanthanide fluorescence immunoassay; GSP, genetic screening processor; GA, gestational age; PPV, positive predictive value; DBS, dried blood spot; BW, birth weight; LC-MS/MS, liquid chromatography-tandem mass spectrometry; MLPA, multiplex ligation-dependent probe amplification; CI, confidence interval
