# Supplementary material for: Neonatal Screening for Congenital Adrenal Hyperplasia in Guangzhou: 7 Years of Experience
Source: Int J Neonatal Screen. 2025 Dec 17;11(4):116. doi: 10.3390/ijns11040116 (PMC12733672; doi:10.3390/ijns11040116)
Supplement: Supplementary file 1 [file IJNS-11-00116-s001.zip › IJNS-3967543-supplementary.pdf]

Table S1. Clinical characteristics, laboratory findings, and genotype of CAH confirmed cases (forty neonates diagnosed with classic 21-hydroxylase deficiency through newborn screening for CAH).

No.	Sex	GA(w)	BW(g)	Time of first sample(hours)	17-OHP first sample(nmol/L)	Time of second sample(days)	17-OHP second sample(nmol/L)	Age of diagnosis(days)	Na+(mEq/L)	K+(mEq/L)	Genotype	Phenotype	Ambiguous genitalia	Family history
1	M	33W+6	2630	78	354.2	15	196.7	15	127.8	6.25	p.Ser97fs*12/del E3-E7	SW		
2	M	35W	2830	72	351.6	15	365.7	15	126.1	6.18	p.Ser97fs*12/p.Arg357Trp	SW		
3	M	36W	3500	86	84.2	14	446.4	14	117	7.6	p.Ser97fs*12/p.Ser97fs*12	SW		
4	F	37W	2760	80	235.7	19	316.4	19	130	6.8	NA	SW	Y	
5	M	37W	2890	42	178.0	10	265.1	10	129	6.1	p.Ile173Asn/del E1-E7	SW		
6	M	38W	2950	67	472.0	10	297	10	136	6.18	p.Ser97fs*12/del E1-E4	SW		
7	M	38W	3840	72	>600	9	300	9	131	6.5	NA	SW		
8	F	38W	3250	312	164.5	18	102.1	18	131.3	6.05	p.Ile173Asn/p.Leu308Phefs*6	SW	Y	
9	M	38W	2850	74	103.7	NA	NA	9	134.2	6.37	p.Ile173Asn/p.Gly111Valfs*21	SW		
10	F	38W	3050	96	75.4	NA	NA	10	115.4	7	p.Ser97fs*12/del E1-E7	SW	Y	
11	F	38W+4	3740	31	29.5	10	64.5	15	141.4	3.86	p.Gly111Valfs*21/p.Ile173Asn	SW		
12	F	38W+5	3090	72	266.3	NA	NA	36	138.7	5.82	p.Ser97fs*12/p.Ser97fs*12	SW	Y	
13	M	38W+6	3500	72	37.1	11	164.2	14	135	6.03	p.Ile173Asn/p.Leu308Phefs*6/E6 cluster	SW		
14	M	39W	3900	115	24.5	88	65.4	88	136.3	4.41	p.Ile173Asn/p.Arg357Trp	SW		Y
15	F	39W	3000	57	508.3	NA	NA	14	128	6	p.Ser97fs*12/p.Ser97fs*12	SW	Y	
16	M	39W	3020	59	530.2	11	>600	12	116	7.8	p.Ser97fs*12/p.Ser97fs*12	SW		
17	M	39W	3860	35	31.9	11	25.1	11	134.8	5.45	p.Ile173Asn/p.Ile173Asn	SW		
18	M	39W	3650	70	326.9	9	473.6	9	134.6	5.63	del CYP21A2/p.Ser97fs*12	SW		
19	M	39W	2960	65	336.1	9	373.8	9	134	4.7	p.Ser97fs*12/p.Gln319*	SW		

20	F	39W	3020	37	564.7	7	305	7	133.1	6.3	del E1-E4/del E1-E6	SW	Y	
21	F	39W+3	3500	88	463.7	8	>300	8	141.2	4.87	p.Ser97fs*12/p.Ser97fs*12	SW		
22	M	40W	3580	68	>600	8	>600	8	126	6.29	p.Gln319*/p.Arg357Trp/p.R484Pfs*58	SW		
23	F	40W	3420	70	267.5	8	198.1	8	131.1	6.79	p.Ser97fs*12/p.Arg484Profs*58	SW	Y	
24	M	40W	3000	43	414.2	9	510	9	121	7.26	p.Ser97fs*12/p.Ser97fs*12	SW		
25	M	40W+1	3770	37	428.1	7	208.6	7	130.5	6.3	NA	SW		
26	M	40W+2	3900	848	186.9	NA	NA	22	112	6.65	p.Gln319*/c.292+1G＞A	SW		
27	M	41W	3470	45	110.0	10	71	12	131.5	6.42	p.Ser97fs*12/p.Arg484Profs*58	SW		
28	F	41W+1	3320	26	296.1	7	404.1	7	136	5.17	p.Arg357Trp/p.Arg357Trp	SW		
29	F	37W	3020	57	26.0	16	74.3	16	140	5.95	p.Ser97fs*12/c.65-175dup	SV		
30	F	37W+3	3020	41	17.2	25	91.9	26	140	5	NA	SV		Y
31	F	38W	3160	75	42.6	16	73.5	16	131	6.2	p.Ile173Asn/p.Gly111Valfs*21	SV	Y	
32	F	38W	3140	43	48.7	15	114.3	16	134.3	6.15	p.Ile173Asn/del E1-E7	SV		
33	F	38W+4	3470	72	42.5	13	32.6	17	137.7	4.5	p.Ser97fs*12/p.Ile173Asn	SV	Y	
34	F	39W	3350	69	49.1	15	115	15	136.1	5.66	p.lle173Asn/p.Ser97fs*12	SV		
35	F	39W	2810	49	15.9	14	80.7	15	139.6	5.88	p.Ile173Asn/p.Met257Arg	SV		
36	F	39W	2850	54	37.9	19	116.8	19	131	6.3	del E1-E3/p.G425S/p.G425S	SV		Y
37	M	39W	3600	49	68.4	12	60.8	12	135.1	5.53	p.Ile173Asn/del E1-E6	SV		
38	F	39W+3	3210	35	23.6	42	360.1	42	132.6	5.85	p.Ile173Asn/p.Arg357Trp	SV	Y	
39	M	40W	3200	74	252.5	15	322.2	15	135.7	5.07	p.Ser97fs*12/p.Ser97fs*12	SV		Y
40	F	40W	3430	81	15.3	16	113.2	19	136.2	4.27	p.Ile173Asn/p.Pro460Leu	SV		

17-OHP, 17-hydroxyprogesterone; BW, birth weight; F, female; M, male; NA: not available; SV, simple virilizing; SW, salt wasting; The data presented in the table are organized according to ascending order of both phenotypic severity and gestational age. Patient 4 in the table died on the 40th day of life.

Table S2. Clinical characteristics, laboratory findings, and genotype of CAH confirmed cases (ten cases of children who did not undergo neonatal CAH screening and three cases of children who were not within the target population for screening).

**No.**	**Sex**	**GA(w)**	**BW(g)**	**Time of first sample(hours)**	**17-OHP first sample(nmol/L)**	**Time of second sample(days)**	**17-OHP second sample(nmol/L)**	**Age of diagnosis(days)**	**Na+(mEq/L)**	**K+(mEq/L)**	**Genotype**	**Phenotype**	**Ambiguous genitalia**	**Family history**	**CAH neonatal screening**
1	F	34W	1600	91	192.5	NA	NA	8	131	6.5	NA	SW	Y		
2	F	37W	3360	55	133.1	33	>600	33	112	5.85	p.Leu262Pro/del E1-E3	SW	Y		
3	M	38W	2890	79	>600	NA	NA	15	138.1	5.11	p.Ser97fs*12/p.Gln319*	SW			
4	F	39W	3800	108	258.5	26	>600	27	137.6	4.81	p.Ser97fs*12/c.1454_1455delGG	SW	Y		
5	M	39W	3150	74	333.6	8	583.7	10	124	5.8	p.Ser97fs*12/del E1-E3	SW			
6	F	39W	2890	80	325.4	31	531.4	31	138.1	5.3	p.Ser97fs*12/del CYP21A2	SW	Y		
7	M	39W+3	3550	90	NA	28	300	28	135.8	4.98	p.Ser97fs*12/E6 cluster	SW			
8	F	39W+4	3150	72	98.1	NA	NA	9	130.6	6.25	p.Leu108Arg/p.Gln319*	SW	Y		
9	F	41W	3020	35	141.1	NA	NA	36	135.4	4.64	p.Ser97fs*12/p.Arg357Trp	SW	Y		
10	F	39W+3	2950	72	419.8	NA	NA	10	100	6.3	p.Arg357Trp/E6 cluster	SV	Y	Y	
11	M	39W	3420	28	35.2	22	8.4	690	136.6	4.56	p.Ser97fs*12/c.-113G>A	NC			Y
12	F	40W	2500	72	15.2	15	8.3	300	136.1	4.8	p.Ser97fs*12/p.Val282Leu	NC			Y
13	M	37W	3580	76	93.9	8	124.1	8	132.7	5.01	HSD3β2 p.Thr259Met/p.Val225Asp	3β-SW	hypospadias	Y	Y

17-OHP, 17-hydroxyprogesterone; BW, birth weight; F, female; M, male; NA: not available; SV, simple virilizing; SW, salt wasting; NC, non-classic. For cases 1－10, neonatal screening for CAH had not been performed; the 17-OHP concentration in the first sample was obtained through reanalysis of the original dried blood spot specimen collected during neonatal screening, following the diagnosis of CAH. The data presented in the table are organized according to ascending order of both phenotypic severity and gestational age.
